# Veterinary Medical Society of the University of Pennsylvania

**Published:** 1897-02

**Authors:** John E. Spindler

**Affiliations:** Secretary


### VETERINARY MEDICAL SOCIETY OF THE UNIVERSITY OF PENNSYLVANIA.

AT the last regular meeting of this Society, held in the lower lecture-room on Friday evening, January 15, 1897, there was a very interesting address on the study of veterinary medicine and the duties of the veterinarian of today and of the future, by our genial friend, Dr. W. Horace Hoskins, a prominent veterinarian of this city. After the address there was an election of officers, which resulted in the electing of the following: President, Louis A. Klein, class of '97; Vice-President, Edward M. Ranck, class of '97; Treasurer, James Beatty, class of '97; Librarian, Stephen Blount, class of '98. Executive Committee: William G. Shaw, class of '97; Ernest P. Spaeth, class of '98; Bassett Kirby, class of '98; and Clinton F. Keiter, class of '99.

The Secretary read the report of the retiring Treasurer, as follows: receipts, $57.77; expenditures, $9.20; balance on hand, $48.57. Signed, Mr. H. Bower, Treasurer. The Secretary reported the receipts of the evening to be $3.50.

After other business of minor importance, the meeting adjourned at 10 P.M.

JOHN E. SPINDLER,
Secretary.

